# Simultaneous Incidental Parathyroid Carcinoma and Intrathyroid Parathyroid Gland in Suspected Renal Failure Induced Hyperparathyroidism

**DOI:** 10.1055/s-0037-1599072

**Published:** 2017-02-28

**Authors:** Andrew Pappa, Trevor Hackman

**Affiliations:** 1 Department of Otolaryngology - Head and Neck Surgery, University of North Carolina at Chapel Hill, Chapel Hill, North Carolina

**Keywords:** tertiary hyperparathyroidism, parathyroid cancer, intrathryoidal parathyroid, intraoperative PTH, parathyroidectomy

## Abstract

Hyperparathyroidism is a common disorder affecting more than hundreds of thousands of people annually. While most commonly secondary to an adenoma, it may also arise from four-gland hyperplasia or malignancy. In the case of primary hyperparathyroidism, the number of glands involved may be unknown prior to surgery. In contrast, the metabolic disorder associated with renal failure induced hyperparathyroidism ensures a hyperplasia picture. Despite the uniform hyperplasia seen in tertiary disease and the preoperative expectation for four-gland exploration, our case demonstrates the continued need for a surgeon's vigilance during dissection to identify all glands and appropriately use intraoperative parathyroid hormone (PTH) testing. In addition, while intraoperative PTH assessment is an effective method for confirming adequacy of treatment for hyperparathyroidism, only surgical pathology can confirm malignancy, which should be considered with PTH levels > 1,000. The case also underscores the importance of comprehensive surgery management and mindful interpretation of intraoperative PTH levels in the management of hyperparathyroidism. Standard surgical technique includes complete exploration of the central compartment, and thyroid lobectomy when the aforementioned exploration fails to reveal the necessary parathyroid tissue, especially with a persistently elevated PTH. Without a standardized progressive compartment exploration and judicious use of intraoperative hormone testing, intrathyroidal parathyroid glands can be missed.

In the United States, approximately 100,000 people develop hyperparathyroidism each year. Approximately, 85% of cases are due to parathyroid adenomas, whereas 10 to 20% are due to four-gland hyperplasia, of which many of these cases are associated with renal disease. 1 The following is a report, approved by the University of North Carolina Institutional Review Board, of a patient treated for presumed tertiary hyperparathyroidism, whose operative procedure and pathology report highlight important learning points in the management of patients with hyperparathyroidism.

## Case Report

A 45-year-old African-American male with a history of hypertension, diabetes mellitus, morbid obesity, and end-stage renal disease on dialysis for 4 years with refractory hypercalcemia (Ca: 10.6–11.8 mg/dL over the prior 12 months) despite 3 days per week hemodialysis presented with tertiary hyperparathyroidism refractory to cinacalcet to the otolaryngology clinic in August 2014 for planned parathyroidectomy for the presumed four-gland disease. He had undergone a left below-knee amputation in January 2014 for progressive peripheral vascular disease and had a history of a CVA (cerebrovascular accident) 2 years prior. He had a calcium level at the time of evaluation in clinic of 9.8 mg/dL, which corrected to 10.6 mg/dL (albumin level 3.0), and a parathyroid hormone (PTH) level of 1,422 pg/mL. In clinic, ultrasonography was performed and demonstrated two distinct hypoechoic round, approximately 1 cm in size, left central compartment masses presumed to be the inferior and superior parathyroids, as well as a large dumbbell-shaped 3-cm right central compartment mass medial to the carotid, presumed to be the juxtaposed right inferior and superior parathyroid glands. No other imaging was performed, as he was presumed to have renal hyperplasia. There was no family history of parathyroid disease.

He underwent an outpatient parathyroidectomy uneventfully in November 2014. Pre-incision PTH was 1,315 ng/mL. The patient's enlarged left superior parathyroid was identified and removed, but no inferior parathyroid gland was seen. The right the superior and inferior parathyroids were identified. The enlarged 3-cm right superior parathyroid was removed, as well as 90% of the inferior gland, with care taken to maintain the remnant gland on its vascular pedicle. Twenty minutes following this resection, the PTH level was 179 pg/mL. Due to the persistent PTH elevation, despite the marked drop from the preincision level, and the lack of an observed left inferior parathyroid, the left thyroid lobe was removed, 10 minutes after which the intraoperative PTH level decreased to 23 pg/mL.

The final pathology report revealed hypercellular parathyroid tissue in the left superior and right inferior parathyroid glands. The right superior parathyroid was involved by a 3-cm parathyroid carcinoma weighing 3.4 g with lymphovascular space invasion. Histologically, the lesion displayed fibrosis without necrosis, and vascular invasion with frequent mitotic figures, P63 positive and calcitonin negative. Vascular invasion was seen within the gland and extending out of the capsule. Lastly, the left thyroid lobe was found to have benign intrathyroidal hypercellular parathyroid tissue. The patient was notified of the results and referred to a radiation oncologist for adjuvant radiation therapy. He remains disease-free today.

## Discussion

Our case demonstrates multiple teaching points with respect to the management of hyperparathyroidism and unique pathological features that practitioners should be aware during the treatment of parathyroid disease, specifically parathyroid carcinoma and intrathyroidal parathyroid glands.

The prevalence of parathyroid carcinoma is remote, thought to be 0.005%, 2 and the incidence of an intrathyroidal parathyroid is between 1.3 and 6.7%, usually within the lower lateral quadrant of the thyroid on the ipsilateral side of the gland. 3 The presence of this rare carcinoma is even less likely in the setting of renal hyperplasia and therefore could have easily been missed without surgical pathology. While the patient's markedly elevated PTH level of 1,422 pg/mL raised our index of suspicion for the possibility of carcinoma, there is no absolute PTH level cutoff differentiating between adenoma and carcinoma. 4 Therefore, while intraoperative PTH assessment is an effective method for confirming adequacy of treatment for hyperparathyroidism, this case demonstrates the underlying importance of a final surgical pathology assessment of the resected specimens, particularly in unusual cases. In our patient's case, the final pathology provided new prognostic information as well as the opportunity for our patient to consider the option adjuvant radiation therapy, which he otherwise would not have had with use of intraoperative PTH levels alone. In addition, while serum calcium levels are typical much higher in parathyroid cancer patients, our patient was receiving 3 day per week hemodialysis. Thus, we surmise his dialysis provided a relative reduction in his serum calcium levels from what would normally be seen with parathyroid cancer to that consistent with a tertiary hypercalcemia patient.

The case also underscores the importance of comprehensive surgery as well as the importance of understanding and using intraoperative PTH levels in the management of these patients. Standard surgical technique includes a complete exploration of the central compartment, including the retroesophageal and retrocarotid space, for parathyroid glands, and thyroid lobectomy when the exploration fails to reveal parathyroid tissue, especially in the setting of elevated PTH. Rajaei et al followed PTH levels in 1,371 patients following parathyroidectomy, finding that patients with final intraoperative PTH levels of <40 pg/mL versus >60 pg/mL were more likely to be disease-free at 5 years, 95.7 versus 74.8%, respectively. 5 In our case, after identifying only three parathyroid glands following complete left central compartment exploration, and an initial 20-minute postresection intraoperative PTH level returned 179 PTH pg/mL, following this guideline, our index of suspicion for an intrathyroidal parathyroid gland was high. This led to our decision to perform a left thyroid lobectomy, which revealed the intrathyroid parathyroid, allowing for a successful completion of the case, marked by a final PTH level of 23 pg/mL.

## Conclusion

While the vast majority of patients with hyperparathyroidism are routinely managed with resection of a parathyroid adenoma or standard four-gland dissection, this case highlights the importance of surgical vigilance.
